# Bassetto et al. reply

**DOI:** 10.1038/s41586-024-07321-3

**Published:** 2024-05-01

**Authors:** Marco Bassetto, Thomas Reichl, Dmitry Kobylkov, Daniel R. Kattnig, Michael Winklhofer, P. J. Hore, Henrik Mouritsen

**Affiliations:** 1 Department of Chemistry, Physical & Theoretical Chemistry Laboratory, University of Oxford, Oxford, UK; 2 AG Neurosensory Sciences/Animal Navigation, Institut für Biologie und Umweltwissenschaften, Carl von Ossietzky Universität Oldenburg, Oldenburg, Germany; 3 Center for Mind/Brain Science, University of Trento, Rovereto, Italy; 4 Living Systems Institute, University of Exeter, Exeter, UK; 5 Department of Physics, University of Exeter, Exeter, UK; 6 AG Sensory Biology of Animals, Institut für Biologie und Umweltwissenschaften, Carl von Ossietzky Universität Oldenburg, Oldenburg, Germany; 7 Research Center for Neurosensory Sciences, University of Oldenburg, Oldenburg, Germany

replying to: S. M. Reppert *Nature* 10.1038/s41586-024-07319-x (2024)

replying to: C. P. Kyriacou *Nature* 10.1038/s41586-024-07320-4 (2024)

We welcome the opportunity to respond to two Comments^1,2^ on our study^3^ of the behaviour of fruit flies in magnetic fields. In Bassetto et al.^3^, we attempted to implement the assays of Gegear and colleagues^4–6^ and Fedele et al.^7^, hoping that they would allow us to use *Drosophila* as a model organism for determining the biophysical mechanisms, genetic basis and neuronal pathways by which animals respond to magnetic stimuli. This proved to be a fruitless endeavour.

Our conclusion^3^ that the magnetic field effects reported by Gegear et al.^4^ were most likely false positives was based on the incorrect choice of statistical tests by these authors. We have discussed these matters extensively in the Supplementary Information of our paper^3^ and in ref. ^8^. Here we provide only a short summary.

The Student’s *t*-test (and similarly analysis of variance) proposed by Krashes and Waddell^9^ and used by Gegear et al.^4^ to analyse group T-maze data is strongly affected by pseudo-replication and is fundamentally wrong for analysing preference indices^3,8^. This is reflected in the exaggeratedly significant results (*P* < 0.0001) claimed in ref. ^4^ for small proportion contrasts (45% naive versus 55% trained). We therefore chose a correct statistical framework for proportions and avoided pseudo-replication by taking each batch of flies as the independent statistical replication unit (biological replicate). This correction, albeit conservative, nonetheless yielded significant results in our positive control experiment (odour-conditioned flies). Reppert^2^ includes additional statistical tests in his Comment (ordinal logistic fit model and Wilcoxon rank sum test) and presents results derived from synthetic data. Ignoring the pseudo-replicative nature of the data, these tests suffer from the same problem as the *t*-test (see above). Statistical tests are based on frameworks of assumptions that are appropriate for specific problems and data structures and cannot be applied out of context. We cannot comment further on these analyses because neither the original data^4^ nor the new synthetic data^2^ have been made available.

Reppert^2^ also writes that the fact that we did not attempt to replicate the negative geotaxis experiments of Bae et al.^10^, together with our failure to reproduce the findings of Fedele et al.^7^, makes our study^3^ an outlier. Once again, this opinion ignores the fact that these studies used statistical approaches that are not appropriate for proportions and therefore led to highly exaggerated *P* values for small to moderate proportion contrasts. Both Fedele et al.^7^ and Kyriacou^1^ (Fig. 1b ^1^) attach inappropriate and deceptive s.e.m.-based error bars to the proportion of non-climbers and thus greatly underestimate the uncertainty in the proportions due to pseudo-replication. Applying the *t*-test to proportions implicitly assumes that each fly in a batch is an independent biological replicate in the extremely strict sense that the decision of each individual was interrogated independently of the other flies (that is, as if each fly were tested individually)^8^. It is clear that the use of an intrinsically pseudo-replicative rapid group assay makes it impossible to know how many flies in a batch made a decision independently of the others (see also Mora et al.^11^). This is why we consider a batch of flies, rather than an individual fly, to be an independent biological replicate. In conclusion, a larger number of studies using invalid statistics does not make them more convincing.

Kyriacou^1^ reanalyses selected parts of our negative geotaxis data, reaching different conclusions. As in Fedele et al.^7^, the first part of his reanalysis is based on an arbitrary criterion as to the definition of climbers, without providing his own data to demonstrate that a group of flies is clearly separable into climbers versus non-climbers and that this categorization can be consistently observed in repeat trials on the same group. As demonstrated in Bassetto et al.^3^, the distributions of heights climbed do not show the bimodal structure that would be required for the approach of Fedele et al.^7^ to be valid^8^. The second part of Kyriacou’s reanalysis deals with the mean height climbed in a given time. For example, his Fig. 1e^1^ hints at a magnetic field effect at 0 μT relative to 90-µT, 220-µT or 300-µT exposures. However, Kyriacou’s analysis^1^ showed no effect for true magnetic field exposure versus sham exposure, the latter being the negative control matched to each magnetic field exposure, with the same currents flowing through the coils as in the magnetic field exposure, but in antiparallel directions, thereby cancelling the coil field and leaving the ambient field. Similarly, the interaction between exposure and exposure intensity was not significant either. Overall, this tallies with our analysis (Supplementary Table 10 in Bassetto et al.^3^), reporting no effects other than occasional random fluctuations, which are expected when comparing many conditions. Similarly, when Kyriacou^1^ chooses a single time point (again the arbitrary 15 s) in a single dataset (Fig. 1f,g^1^ for CS-OX in the FlyVac setup at 300 µT), one may see a small effect, but not when taking into account the full time-dependence of the climbing behaviour (Supplementary Table 11 in Bassetto et al.^3^). Thus, Kyriacou’s Comment^1^ focuses on an outlier confined to an arbitrary time point in a single exposure condition. By contrast, our statistical analyses took into consideration all of the experimental conditions together with the complete climbing performance. It is clear that any experiment has outliers, the absence of which would be suspicious. Critically, an outlier cannot be taken as an effect once the exploratory data analysis has already been carried out (a practice known as HARK-ing—hypothesizing after the results are known), but can serve only as the basis of a test hypothesis to be confirmed or rejected in a repeat experiment.

Kyriacou^1^ remarks on the accuracy of the video tracking of fly movements in our negative geotaxis experiments^3^. The number of frames logged is simply a result of flies being out of bounds. Frames in which flies were hidden by the stoppers at the top or the supports at the base of the tubes were not included in the analysis. We also did not log flies that had arrived at the top of the tubes and had started to descend. This does not imply that flies were not tracked while they were climbing. Moreover, it is not necessary to track a fly in every frame to determine its climbing rate. By contrast, Fedele et al.^7^ simply reported the proportion of flies that climbed to an arbitrarily chosen height within an arbitrarily chosen time period with no further data or photographic documentation.

Kyriacou^1^ suggests that the absence of a magnetic response in our direct replication of Fedele et al.^7^ (using the original equipment he had kindly loaned us) was due to our low blue-light intensity’s resulting in small differences in geotactic responses between red- and blue-light conditions. As mentioned in Bassetto et al.^3^, we used the same intensity (0.25 μW cm^−2^) as Fedele et al.^7^. The *P* value for the effect of the wavelength of the light on climbing performance was very highly significant for the CS-OX strain^3^ and just significant for the CS-LE flies^3^. We agree that the CS-LE strain received from Kyriacou may not have been ideal, but the CS-OX strain clearly passed the control.

Kyriacou^1^ wonders why we used magnetic fields weaker than those of Fedele et al.^7^ (500 μT) in some of our experiments. In our exact replication of Fedele et al.^7^, with Kyriacou’s original apparatus, we used 500 μT. Having failed to find a magnetic response under those conditions, we used two improved experimental designs (gravity and FlyVac assays)^3^ with much more homogeneous magnetic fields of up to 300 μT. The radical pair mechanism^12^ provides no theoretical reason to expect a large difference in the responses to such similar field strengths.

Reppert^2^ berates us for conducting the positive conditioning (olfactory) controls and the magnetic exposure experiments in different locations, claiming that sugar-reinforced conditioning is a complex behavioural paradigm, sensitive to temperature and humidity. There is no evidence in Gegear et al.^4^ to support such a contention. We chose to carry out the olfactory controls^3^ in Scott Waddell’s laboratory in Oxford specifically to take advantage of his facilities and expertise working with odour stimuli. For similar reasons, we carried out all of the magnetic stimulus tests^3^ in Oldenburg, where the experimental facilities for controlling magnetic fields are second to none^13,14^.

Reppert^2^ is concerned about our comments on the sample sizes used by Gegear et al.^4^ made in the context of the inappropriate statistical methods used by these authors (see above). The numbers are as follows. For the wild-type Canton-S flies, which showed the strongest magnetic responses reported in their paper, Gegear et al.^4^ (Fig. 1b^4^) studied 22 groups of 100–150 flies (12 trained, 10 naive), whereas Bassetto et al.^3^ (Fig. 1a,b^3^) used 300 groups of about 100 flies (50 trained and 100 naive for each of the OX and LE wild-type strains). In this key experiment, using an order of magnitude more samples, we failed to find a magnetic field effect^3^ even when we used the inappropriate statistical analysis used by Gegear et al.^4^.

Reppert^2^ regrets that we did not consider the monarch butterfly as a model organism for studying the mechanism of light-dependent magnetoreception. Given the reports that monarchs should be able to orient in the Earth’s magnetic field (reviewed in ref. ^15^), these genetically tractable insects could be a potential alternative to *Drosophila*. However, in two separate studies^16,17^, we have found no evidence that monarchs have such an ability: 140 migratory monarch butterflies tested with access to only natural geomagnetic field cues showed random orientation, whereas monarchs tested with celestial cues showed a clearly directed southwest orientation^16^. Furthermore, monarchs first flown in the normal magnetic field did not react to a horizontal 120° turn of the field even when they were kept flying in the rotated field for up to 2 h (ref. ^17^).

Independent replication of experimental data is the ‘gold standard’ in science. Meticulously carried out replication studies that fail to confirm earlier results are just as important for the integrity of knowledge as those that do. We suspect that many negative replication attempts are never published. Authors can be reluctant to write them up (and some editors to publish them), resulting in an unbalanced body of literature. We encourage anyone who has tried and failed to observe *Drosophila* magnetoreception to submit their findings to reputable journals.
